# Challenges in the Oral Administration of Gastro-Resistant Formulations: The Role of Vehicles and Bottled Waters

**DOI:** 10.3390/pharmaceutics18040453

**Published:** 2026-04-08

**Authors:** Adrienn Katalin Demeter, Dóra Farkas, Márton Király, Ádám Tibor Barna, Krisztina Ludányi, István Antal, Nikolett Kállai-Szabó

**Affiliations:** 1 Department of Pharmaceutics, Faculty of Pharmacy, Semmelweis University, Hőgyes E. Street 7-9, 1092 Budapest, Hungary; demeter.adrienn.katalin@semmelweis.hu (A.K.D.); farkas.dora@semmelweis.hu (D.F.); kiraly.marton@semmelweis.hu (M.K.); barna.adam@semmelweis.hu (Á.T.B.); ludanyi.krisztina@semmelweis.hu (K.L.); antal.istvan@semmelweis.hu (I.A.); 2 Center for Pharmacology and Drug Research & Development, Semmelweis University, Üllői Street 26, 1085 Budapest, Hungary

**Keywords:** gastro-resistant, pellets, multiparticulate system, method of manipulation, drug administration, dysphagia, bottled water

## Abstract

**Background/Objectives**: Gastro-resistant multiparticulate systems are designed to protect drugs in acidic environments and to ensure intestinal release. In practice, the method of administration may need to be modified: pellet-containing capsules opened or tablets halved for patients with swallowing difficulties, yet the type of liquid used for administration is often not specified. This study examined the stability of gastro-resistant coated pellets after exposure to various aqueous media prior to ingestion. **Methods**: To evaluate administration instructions, 103 Summaries of Product Characteristics of gastro-resistant products were reviewed. Pellets were produced using a bottom-spray fluidized bed process and coated with Eudragit L 30 D-55. Dissolution testing in pH 1.2 medium was performed after pre-soaking the pellets for 5, 15, and 30 min in beverages with various pH and conductivity. Drug release was measured by UV-VIS method, and morphological changes were assessed by image analysis. Marketed gastro-resistant products were also examined visually. **Results**: SmPC review revealed that the beverage for intake was frequently unspecified. Among the tested beverages differences in pH and conductivity were observed. Alkaline medicinal mineral waters induced increased and time-dependent premature drug release compared to tap and filtered water. Image analysis indicated a reduction in surface area after exposure to alkaline media. **Conclusions**: Contact with non-specified aqueous media before swallowing may weaken the protective function of gastro-resistant films. More explicit recommendations on suitable administration manipulation and media may improve therapeutic consistency.

## 1. Introduction

Oral administration remains the most common route for drug delivery due to its convenience and high patient compliance. In recent decades, pellet-based multiparticulate systems have become an essential focus of pharmaceutical research [1]. Their distinct technological and biopharmaceutical advantages over single-unit dosage forms make them highly valuable in both formulation design and drug development [2,3]. The pellets containing active pharmaceutical ingredient(s) (API) can be used for multiparticulate systems, or, in other words, multiple-unit pellet systems (MUPS) [4]. The presence of the pellets within a single dosage form, for example, filled in a capsule [5] or compressed in a tablet, provides several technological, physiological, and even therapeutic advantages [6,7].

Pellets and other multiparticulate systems are designed to divide the dose of API into discrete subunits, facilitating more predictable transit through the gastrointestinal tract and reducing the risk of dose dumping or local irritation [8]. Their regular spherical shape and favorable size distribution enable efficient processing, coating, and blending of different drug substances, thereby providing greater flexibility in formulation design [9,10]. Multiparticulate systems can be produced using various manufacturing techniques, each with specific process conditions that result in products with distinct physicochemical characteristics. From the point of view of the API amount distribution, the conventional pellet can be matrix (homogeneous) or layered (heterogeneous) [11,12].

One way to modify the drug-release profile is by coating. Coating of multiparticulate systems may be performed to achieve specific functional properties, improve chemical stability, optimize physical characteristics, or enhance patient acceptability [13,14]. Functional coatings, such as films that dissolve in the intestine or stomach, are widely used in oral drug delivery to protect APIs from degradation in the stomach, to control the location and rate of drug release. Enteric coatings remain intact in the acidic gastric environment (pH 1–3) and dissolve at higher pH values in the small intestine (pH 5.5–7.5), facilitating targeted delivery and reducing drug degradation in the stomach [15,16]. This protective mechanism is achieved by ionizing carboxylic acid groups, thereby allowing the polymer to dissolve. Anionic methacrylate copolymers, such as Eudragit L 30 D-55, are among the most commonly used pH-dependent gastro-resistant materials due to their predictable dissolution behavior at specified pH and suitability for aqueous coating processes [17,18]. Gastro-resistant polymers have been widely used in multiparticulate dosage forms to improve stability in the stomach and modulate the in vitro drug release profile, even when the administration method is altered [19].

Solid oral dosage forms, such as capsules and tablets, are still widely used, despite a significant percentage of the population having difficulty swallowing [20,21,22]. These patients with dysphagia who do not follow recommendations in the prescriptions may not receive the maximum benefit of the medications [23,24]. In case of some drugs, manipulating the administration form is permitted; however, inappropriate medication use can alter the drug’s pharmacokinetics and pharmacodynamics and compromise treatment efficacy and patient safety [25]. To overcome swallowing difficulties in patients with dysphagia, innovative film-coating materials can be used [26], or medications can be split and administered with soft foods or other vehicles, or delivered via feeding tubes [27,28], especially for the elderly [29,30].

Whenever marketed products are manipulated (capsules opened, tablets halved, etc.), concerns about achieving the required API dose arise, and the method of manipulation is also essential [31]. Patients can use non-standard vehicles to take their medicines, although standardized pharmacopeial media are typically used for dissolution testing [32,33,34]. In most cases, patients are advised to take their oral drugs with liquid; however, it is not certain which vehicles the patient will choose. Although some Summary of Product Characteristics (SmPCs) allow the method of intake to be manipulated, the type of liquid used for dispersion is often not explicitly specified. The vehicles can be different kinds of water, cola, beer, or milk, and the choice can alter the medication [35]. Research suggests that people choose between tap water and bottled water mostly based on taste, sensory perception, and perceived water quality. Certain groups (such as children) are more likely to prefer bottled water, especially if they have had previous negative experiences with the quality or taste of tap water [36,37]. 

Maintaining the integrity of the enteric coating is critical, particularly for acid-labile drugs [38] whose therapeutic efficacy depends entirely on protection from gastric degradation. Premature coating failure may result in drug degradation in the stomach, subtherapeutic plasma concentrations, treatment failure, or, in some cases, increased gastric side effects, with potentially serious consequences for patient safety and treatment outcomes.

These considerations highlight that, in the context of oral administration, the vehicle used during ingestion may influence not only the active pharmaceutical ingredient (e.g., ciplofloxacin in milk) but also the used excipients, and that impairment of excipient function may in turn compromise the overall performance of the dosage form. Accordingly, this study reviewed patient information leaflets (PILs) and Summary of Product Characteristics (SmPCs) of 103 gastro-resistant medicines to assess the prevalence of non-specific guidance, characterized commonly consumed beverages to identify conditions relevant to gastro-resistant coatings, and evaluated the effects of selected media on marketed products. Finally, a model pellet containing a suitable drug (caffeine) with a gastro-resistant coating was formulated to quantitatively investigate the impact of pre-soaking duration and fluid type on drug release.

## 2. Materials and Methods

### 2.1. Materials

For pellet production, inert microcrystalline cellulose cores were used: Cellets 780 in the size range of 710 μm–850 μm (Process-Center GmbH & Co. KG, Dresden, Germany). The active ingredient of the pellets was caffeine (Molar Chemicals Kft., Halásztelek, Hungary), as a model drug. Sunset Yellow FCF (303CC Sensient Food Colors UK Ltd., Norfolk, UK) was used as the coloring agent for the drug-containing layer. Hydroxypropyl methylcellulose (HPMC; Pharmacoat 606, Shin-Etsu Chemical Ltd., Tokyo, Japan) was used as the binder excipient during the drug layering process. Eudragit L 30 D-55 (Evonik Industries AG, Essen, Germany), a methacryl-ethyl-acrylate copolymer, was used to formulate the release-modifying coating. Micronized talc powder (Harke Pharma GmbH, Mülheim, Germany) was incorporated into the film-coating dispersion to improve adhesion. Triethyl citrate (TEC; Sigma-Aldrich Chemie GmbH, Darmstadt, Germany) served as the plasticiser for the film coating.

For the dissolution studies, different kinds of liquids were used: Peridot (Peridot Aqua Kft., Budapest, Hungary), Salvus (Salvus Kft., Pilisszentiván, Hungary), Parádi (Medaqua Kft., Gyöngyös, Hungary), and Hunyadi (Medaqua Kft., Gyöngyös, Hungary) bottled waters, apple juice (Sonny Kft., Budapest, Hungary), filtered water (Culligan Hungary Kft., Budapest, Hungary), and pH 1.2 ± 0.05 hydrochloric acid medium. For further examination, the liquid abbreviations are listed in Table 1.

### 2.2. Preliminary Review of the SmPC Provisions

A preliminary review of the SmPCs (as well as Patient Information Leaflet) was conducted to assess how the oral route of administration is addressed in official documentation; the search focused on gastro-resistant products. To evaluate the relevance of this issue, the official Hungarian medicinal database was screened using the keyword “gastro-resistant” [39]. Only products registered with an active status on 2 February 2026 were included, with each product counted as one with the same name but different doses.

### 2.3. Preliminary Studies of the Vehicles

A broad preliminary screening of 22 beverages was conducted to characterize the range of pH and conductivity values among possible vehicles for medicine intake, which are commonly consumed by patients. The results of this screening show the selection of representative test media for the subsequent examination of marketed products and dissolution studies.

The chosen vehicles for this experiment are listed in Table 2 below.

The pH meter (Seven Compact S220, Mettler-Toledo Kft., Budapest, Hungary) was calibrated using buffer solutions of known hydrogen-ion activity. The glass electrode was immersed in 50 mL of the solution, and pH measurements were performed in three parallel experiments at room temperature. For the conductivity measurements, a conductometer (Crison EC-Meter BASIC 30, Barcelona, Spain) was also used in three parallel measurements at room temperature.

After pH and conductivity measurements, the 7 most relevant vehicles were selected, and viscosity was also measured for these. The viscosity of the seven drinks was measured using a Fluidicam^TM^ RHEO viscosimeter (Microtrac Formulaction, Toulouse, France), which is based on microfluidic principles, and can be used to determine the viscosity of various liquids and semi-solid formulations, including gels [40]. An appropriate protocol was chosen: the temperature was set to 25 °C, a 150 μm plastic chip was used, the shear rate was set to 2000–500 s^−1^, 5 points per curve were taken, and each point was calculated from 10 measurements. The proper reference solution was chosen, which, for the beverages, was measured at 5 mPa·s.

### 2.4. Examination of Marketed Products

Commercially available gastro-resistant pellet-containing capsule dosage forms were selected as model products. The chosen products were: Emozul 20 mg, Lansoptol 15 mg (KRKA, Novo Mesto, Slovenia), Emillan 15 mg (PharmaSwiss, Prague, Czech Republic), Ludea 20 mg (Richter Gedeon Nyrt., Budapest, Hungary), Omeprazol-Teva 20 mg (Teva Gyógyszergyár Zrt., Debrecen, Hungary). The coating of the preparations is methacrylic-ethyl acrylate copolymer (1:1), except for Lansoptol. In this preparation, the coating is methacrylic acid methyl methacrylate copolymer (1:1) (Eudragit L 100) and methacrylic acid methyl methacrylate copolymer (1:2) (Eudragit S 100).

As the instructions allowed, the capsules were opened, and the enclosed pellets were examined. The capsules were emptied into a half-cup of liquid (120 mL) for further examination within 30 min, as most SmPCs allow this. During and after soaking, the pellets were visually examined. The study media were tap water, Salvus medicinal mineral water (SMM), and Peridot natural mineral water (PNM). Tap water was included as it is commonly used and suitable for drinking in Hungary, and it serves as a neutral medium commonly used for medication administration. SMM and PNM water were selected as representative examples of commercially available medicinal mineral and natural mineral waters that patients may consume to relieve gastric hyperacidity.

The active ingredients in these preparations all belong to the proton pump inhibitor (PPI) class, and their stability is particularly low. To avoid premature activation in the stomach after oral administration, they must be protected from gastric acid, e.g., with enteric coating. PPIs are absorbed in the duodenum [41].

### 2.5. Formulation of the Pellets

#### 2.5.1. Drug Layering on Inert Pellet Cores

Drug layering on the microcrystalline cellulose inert pellet core was carried out using the fluidized bed bottom-spray method in an Aeromatic STREA-I laboratory fluidized bed system (Aeromatic-Fielder AG, Bubendorf, Switzerland) [42].

Caffeine was selected as a model drug due to its aqueous solubility, well-characterized UV absorbance at 273 nm, and chemical stability across a wide pH range, making it a suitable and widely used model compound for evaluating the protective function of gastro-resistant coatings independently of API-specific degradation effects in the gastric environment. Microcrystalline cellulose spheres were selected as the inert pellet core due to their well-defined spherical geometry, high mechanical strength, and chemical inertness, which ensure that any observed changes in dissolution behavior or coating integrity can be attributed exclusively to the effect of the administration medium on the gastro-resistant film coating, without interference from the core material.

For the first layer of the API (5.0 *w*/*w*%), two solutions were prepared: in half the water (225 mL water), a 4.0 *w*/*w*% aqueous HPMC (9 g HPMC) solution; and in the other half (225 mL water), the caffeine (22.5 g) was dissolved at high temperature due to its solubility. The two solutions were then mixed, and the coloring agent (0.1 *w*/*w*%, 4.5 mg) was dissolved in the media. During the process, the solution was tempered (about 50 °C) to keep the API in solution, but also not to cause the precipitation of HPMC. The endpoint for this part was to achieve 8.5 *w*/*w*% API content. The pellets were then dried at room temperature for at least 24 h.

The formulation parameters are listed in Table 3.

#### 2.5.2. Film Coating

Due to the coatings used in the listed marketed products, Eudragit L 30 D-55 polymer was used for the experiment. Eudragit L 30 D-55 is an aqueous dispersion of a methacrylic acid-ethyl acrylate copolymer (1:1) that remains intact in an acidic medium, providing effective protection against gastric acid, and dissolves rapidly in an alkaline medium, enabling targeted drug release in the proximal small intestine [17].

The gastro-resistant film coating suspension was sprayed onto the drug-layered pellets using a fluidized bed coating process with a bottom-spray configuration. The process parameters are listed in Table 3. During the layering process, the dispersion was gently and continuously stirred and tempered (about 40 °C) to prevent sedimentation of the talc. During the process, samples were taken for further analysis after 10%, 15%, 20%, and 25% weight increases relative to the initial pellet mass.

Table 4 summarizes the exact composition and functions of the coating dispersion used to achieve modified drug release [43].

### 2.6. Examination of the Formulated Pellets

#### 2.6.1. Determination of the API with UV-VIS Spectroscopy

The concentrations of caffeine in the dissolution samples were determined by UV spectrophotometry using an Agilent 8453 UV-VIS spectrophotometer (Agilent Technologies, Waldbronn, Germany) at 273 nm [44,45].

For that, a calibration curve was first made. A stock solution of caffeine was prepared at pH 1.2, and then the calibration standards were diluted from the stock solution (100 µg/mL) to obtain eight calibration levels in µg/mL as follows: 0.1, 1.0, 2.0, 4.0, 6.0, 8.0, 10.0, 15.0. Each standard solution was analyzed at 273 nm with a UV-VIS spectrometer (Agilent Technologies, Waldbronn, Germany). The standard curve was plotted using absorbance and concentration.

Intra-day accuracy and precision were assessed by evaluating five replicates of quality control (QC) samples (*n* = 5). Accuracy was expressed as a percentage of the nominal concentration, and precision was calculated as the relative standard deviation (RSD). The acceptance criteria for both parameters were set at ±5%. The lower limit of quantification (LLOQ) was also determined (*n* = 5) [46]. The ±5% acceptance criterion for intra-day accuracy and precision was selected in accordance with the requirements generally admitted for analytical methods applied to pharmaceutical formulations, where an acceptance limit of 5% is considered appropriate, as opposed to the broader ±15% typically permitted for biological matrices [47]. This criterion reflects the relatively straightforward nature of UV-VIS spectrophotometric quantification of caffeine in a simple aqueous dissolution medium, which is less sensitive to matrix interference and signal variability [48].

The API content of the samples was determined from approximately 100 mg of the pellets with only drug layering, and an average content was calculated. The requirements were set that the API content in 100 mg pellets needs to be between 85 and 115% of the average content; only one can be outside this range, but has to be between 75 and 125% and none are allowed to be outside the limits, according to the most relevant section of the European Pharmacopoeia “2.9.6. Uniformity of content of single-dose preparation” [49]. To determine this, ten parallel measurements were performed.

The dissolution test was carried out by Hanson SR-8 Plus™ Dissolution Test Station (Hanson Research, Los Angeles, CA, USA) employing the rotating basket method (USP 30 Dissolution Apparatus I) at a rotation speed of 100 rpm. Dissolution testing was performed in 900 mL of an acidic medium (pH 1.2), at 37 ± 0.5 °C. The 5 mL samples were manually taken with an automatic pipette at 5, 10, 20, and 30 min, and the media were immediately replaced with the dissolution acidic medium. After sampling, each sample was filtered through a 0.45 μm membrane filter (Nantong Filterbio Membrane Co., Ltd., Nantong City, China).

#### 2.6.2. The Appropriate Gastro-Resistant Coating

Following the drug layering and gastro-resistant film coating, the next important step was to determine the appropriate coating level. The coating level was assessed indirectly by determining the right percentage weight gain relative to the initial pellet mass. For that, the pellets were evaluated using in vitro dissolution testing with the Hanson SR-8 Plus™ Dissolution Test Station (Hanson Research, Los Angeles, CA, USA) employing the rotating basket method at a rotation speed of 100 rpm. Dissolution testing was performed in 900 mL acidic medium (pH 1.2), at 37 ± 0.5 °C. The 5 mL samples were taken with an automatic pipette manually at 15, 30, 45, 60, 90, and 120 min, and the media were immediately replaced with the dissolution acidic medium. After sampling, each sample was filtered through a 0.45 μm membrane filter. For each coating thickness, six parallel measurements were carried out until the correct one was found. The amount of drug released at pH 1.2 over 2 h was determined according to the monograph for gastro-resistant granules, and a release below 10% was used as the acceptance criterion. In accordance with the article on gastro-resistant granules [50], the study duration was set at 2 h.

#### 2.6.3. Image Analysis

The images of the particles (*n* = 100) were made using a Keyence VHX-7000 optical microscope (Keyence Corp., Osaka, Japan) at consistent magnification and underlighting conditions to ensure comparability across all experiments. For higher-contrast images, the glass underlay was used with underlighting to make the outline stand out clearly. This imaging system was used to acquire high-resolution images of the particles, enabling detailed evaluation of their visual attributes, such as size, perimeter, and Feret diameter—for which ImageJ software (Wayne Rasband, National Institute of Health, Bethesda, MD, USA; version 1.54f) was used. In the software, each pellet image was converted to 8-bit grayscale, and then a threshold was set to clearly distinguish the particle boundary from the background. Results were expressed as the mean ± standard deviation across all measured particles and compared between soaking media and timepoints to assess time- and medium-dependent morphological changes.

For quantitative measurements, tap water was selected as the most commonly used medium for medication intake, while PNM water was chosen as a representative alkalizing mineral water that induced progressive, detectable coating degradation without damaging the pellet core, thereby enabling meaningful time-dependent morphological analysis.

The pellets were also inspected individually under a microscope to visually observe the coating degradation process. The most representative ones were selected for demonstration in pH 1.2, tap water, and PNM water.

#### 2.6.4. Dissolution Study

In vitro dissolution studies were conducted to investigate the effect of water pH and ionic composition on the gastro-resistant coating. To quantitatively assess the impact of pre-soaking on gastro-resistant coating integrity, dissolution studies were performed following pre-soaking in seven media selected to cover a wide spectrum of pH and ionic composition identified in the preliminary characterization: Salvus, Parádi, Hunyadi and Peridot water as representatives of alkalizing medicinal and natural mineral waters; tap water and filtered water as representatives of neutral media commonly used for medication intake; and apple juice as a representative acidic beverage.

Dissolution testing was performed in two phases: in the first phase, 100 mg pellets were placed in 120 mL of different liquids for 5, 15, and 30 min at 25 ± 1 °C (room temperature), as a patient would do and as allowed by the SmPCs. For the second phase, after the determined time, to minimize pellet loss and to ensure complete transfer, the media and the pre-soaked pellets were poured into the basket, which was in its initial, raised position. The dissolution vessel below was already filled with 780 mL of 37.0 ± 0.5 °C (body temperature) preheated pH 1.2 medium, so after the transfer, the total volume was 900 mL. The volume of soaking medium transferred (120 mL at 25.0 ± 1.0 °C) relative to the preheated dissolution medium (780 mL at 37.0 ± 0.5 °C) is relatively small, resulting in rapid thermal equilibration upon mixing and minimizing sustained thermal shock. Then, the rotating baskets were lowered into the dissolution medium simultaneously, the rotation was set to 100 rpm, and a timer was started.

5 mL samples were taken with an automatic pipette manually at specified times (5, 10, 15, 30, 60, 90 min), and the medium was immediately replaced with the dissolution pH 1.2 acidic medium. After sampling, each sample was filtered through a 0.45 μm membrane filter. The caffeine concentration was determined with UV-VIS spectrophotometry. For each soaking time and each water type, three parallel measurements were performed to assess differences.

#### 2.6.5. Statistical Analysis

Statistical analysis was performed (R software, version 4.5.3) to evaluate the differences in drug release among the tested administration media. A one-way analysis of variance (ANOVA) was conducted to assess the overall effect of the administration medium on API dissolution. Dunnett’s post hoc test was subsequently applied to compare each medium with tap water as the reference condition, as tap water is the most commonly used liquid for medication intake.

## 3. Results

### 3.1. Results of the Preliminary Review of the SmPC Provisions

A total of 103 SmPCs containing gastro-resistant oral dosage forms were identified and evaluated according to their administration instructions. Several SmPCs allow the mode of administration to be modified, if necessary, such as opening capsules for patients with swallowing difficulties. However, the conditions under which such manipulation may be safely performed are not always explicitly specified, which can result in premature damage to the enteric coating and reduced therapeutic efficacy.

**Figure 1:**
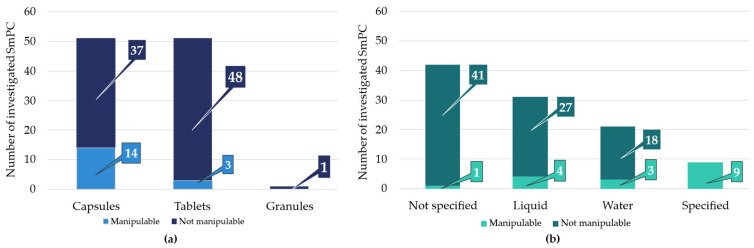
(**a**) Dosage form of the 103 gastro-resistant medicines and (**b**) their administration specifications according to the SmPCs.

The distribution of dosage forms is shown in Figure 1a, which includes 51 capsules (4 soft capsules), 51 tablets, and 1 granule. Among these, manipulation of the dosage form (e.g., splitting tablets or opening capsules) was permitted in a limited number of cases: 14 times for capsules and 3 times for tablets.

Analysis of the administration instructions revealed a wide range of variability in the level of detail provided regarding the liquid used for administration, as shown in Figure 1b. In 42 SmPCs, the vehicle for oral administration was not specified. A total of 31 SmPCs allowed administration with an unspecified fluid, whereas 21 SmPCs explicitly recommended water as a vehicle, but did not specify the type of water. Only 9 SmPCs provided clearly specified administration instructions defining the appropriate liquid to be used, such as apple sauce, sour, soft food/liquid, water, slightly acidic liquid, acidic fruit juice, apple juice, tomato juice, yogurt, and in all 9 cases, the oral dosage form was manipulable.

Further analysis of the SmPCs revealed that the range of active ingredients is quite broad, even within these 103 preparations. According to the ATC (Anatomical Therapeutic Chemical) classification, 52 belong to the group of the medicines for alimentary and metabolism (A), 22 of them belong to antineoplastic and immunomodulating agents (L), 11 of them effect the nervous system (N), 9 of them are for curing blood and blood forming organs and the rest belongs to other groups, as listed in detail in Table 5.

In summary, this structured review reveals that in 94 out of 103 SmPCs, alkalizing media were not explicitly excluded as administration vehicles, manipulation was permitted in only 17 cases, and clearly specified appropriate vehicle guidance was provided in only 9 cases—collectively representing significant and widespread gaps in current product pills for patients that may pose a risk to coating integrity and therapeutic effect in patients requiring dosage form manipulation.

### 3.2. Conductivity, Viscosity and pH Measurement of the Liquids

**Figure 2:**
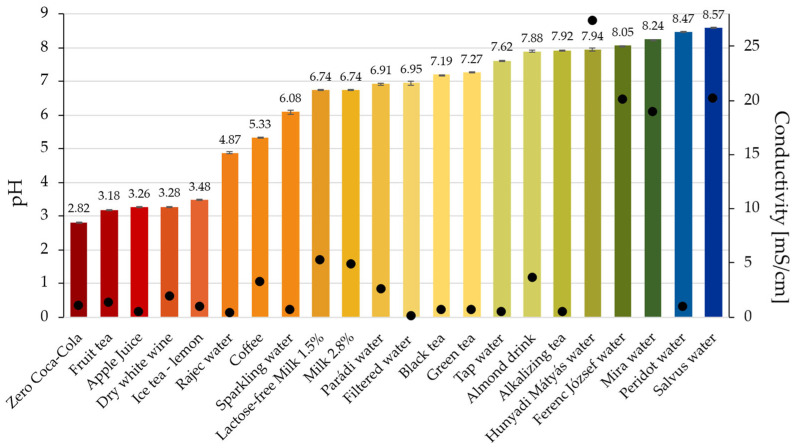
pH (column) and conductivity (dot) of beverages used for administration.

Preliminary characterization of the administered waters and beverages was performed by measuring their pH and electrical conductivity. As shown in Figure 2, the investigated medicinal and natural mineral waters and beverages spanned a wide pH range, from acidic to clearly alkaline.

Electrical conductivity also varied notably among the samples, indicating substantial differences in ionic composition. While several waters exhibited low conductivity, others showed considerably higher conductivity, reflecting higher dissolved ion content. No direct linear relationship was observed between the pH and conductivity across the tested medicinal and natural mineral waters.

These results demonstrate that mineral waters potentially used for medication administration differ significantly in both pH and ionic composition, providing a diverse set of conditions for subsequent evaluation of gastro-resistant coating performance.

The viscosity of the seven vehicles used in the dissolution study was measured across five shear rates ranging from 498 to 2057 s^−1^. As presented in Table 6, all tested media exhibited viscosity values between 0.9 and 1.1 mPa·s across all measured shear rates, which are consistent with the viscosity of pure water at room temperature. No meaningful differences in viscosity were observed among the mineral waters, tap water, and filtered water, which all showed average viscosity values of 0.9–1.0 ± 0.01 mPa·s. Apple juice showed a slightly higher average viscosity of 1.1 ± 0.01 mPa·s. These results confirm that viscosity differences among the tested media were negligible and that the observed differences in dissolution behavior can be attributed primarily to differences in pH and ionic composition rather than to rheological properties of the administration liquids.

### 3.3. Results of the Visual Observations on the Marketed Products and the Formulated Pellets

Overall, distinct differences were observed among the tested medicinal and mineral waters and formulations (commercially available and model-formulated). Salvus (SMM) induced the most visible degradation of the gastro-resistant coating, as opalescence developed within 5 min in all examined products when half a cup of water was used. In the case of Peridot (PNM), a slightly longer exposure was required before marked opalescence became obvious. Based on visual evaluation, product 4 and product 5 were the most resistant to PNM.

When the products were tested in tap water, the majority of formulations maintained coating integrity, and the medium remained visually clear throughout the observation period. However, opalescence appeared in tap water too, for product 3 and product 2, indicating reduced resistance compared to other tested products, and slightly in product 1.

**Figure 3:**
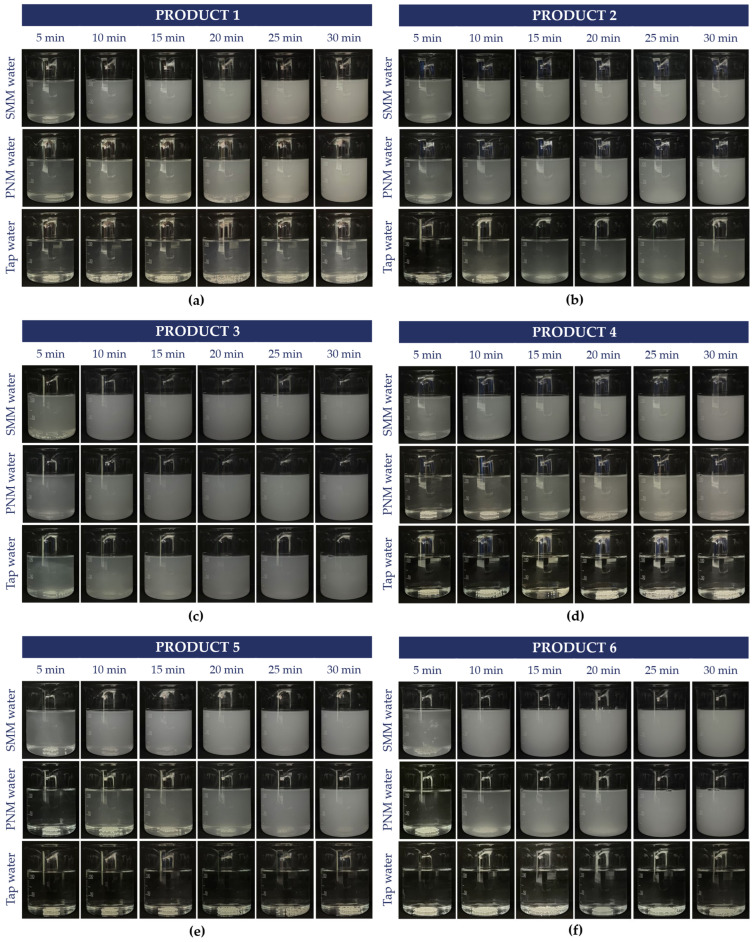
The visual observations of the marketed products in SMM (Salvus medicinal mineral water), PNM (Peridot natural mineral water), and tap water. The tested products: (**a**) product 1, (**b**) product 2, (**c**) product 3, (**d**) product 4, (**e**) product 5, and (**f**) product 6.

The comparison of the six texted products across three media is shown in Figure 3.

**Figure 4:**
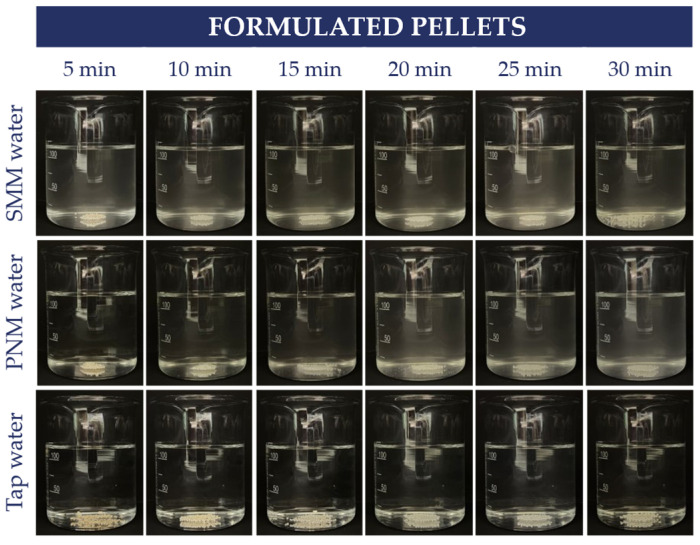
Visual observation of the formulated caffeine pellets in SMM water, PNM water, and tap water.

For comparison, the formulated caffeine pellets were also observed using the same method, as shown in Figure 4. It is visible that the formulated pellets were the most resistant.

### 3.4. The Formulated Pellets’ Test Results

#### 3.4.1. Caffeine Content Determination with UV-VIS

Quantitative determination of the API was performed by UV-VIS spectrophotometry after dissolution of the pellets with only the drug layer. A calibration curve was established using standard solutions prepared over a specified concentration range. The resulting calibration equation demonstrated linearity within the applied range (R^2^ = 0.9997).

The average API content in 100 mg pellets was calculated from the 10 dissolution measurements: 7.53 ± 0.042 mg; the minimum was 7.46 mg, and the maximum was 7.58 mg. The samples meet the pharmacopoeial criteria, with an average API content of 85–115%.

Based on the calibration curve, three QC levels were defined and evaluated. These included concentrations of 0.075 mg/100 mL (LLOQ), 0.10 mg/100 mL, and 0.75 mg/100 mL. The validation parameters for the method are shown in Table 7, and all parameters are within the specified range. Because of the LLOQ, further measurements below 0.075 mg/mL API, or absorbance below 0.0414 ± 0.00134, were excluded.

#### 3.4.2. Determination of the Optimal Thickness of the Gastro-Resistant Coating

Determining the optimal layer thickness for the gastro-resistant coating is essential. With different coating *w*/*w*% weight increases, the samples were tested in order of increasing weight, until the one with the lowest API dissolution (<10%) was found after 2 h at pH 1.2. According to our dissolution testing, the 10 *w*/*w*% was not satisfactory yet; the lowest dissolved caffeine percentage was 13.20%, and the RSD was below 5%. Then, in pellets with the 15 *w*/*w*% weight increase, the gastro-resistant coating was already sufficiently resistant; the highest dissolution percentage was 8.95% from the three parallel measurements.

These results confirm that coating thickness is a critical parameter determining the acid resistance of the enteric film. Therefore, the measurements were continued with pellets that had a 15 *w*/*w*% weight increase gastro-resistant coating.

#### 3.4.3. Results of the Image Analysis

Quantitative image analysis was performed to evaluate potential morphological changes in the pellets after soaking in tap water and Peridot medicinal mineral water. The results are summarized in Table 8.

In tap water, only minor changes were observed over the investigated soaking period (5–30 min). A slight decrease in the area was detected from 0.750 ± 0.6092 mm^2^ to 0.683 ± 0.0798 mm^2^. Similar gradual reductions were observed in the perimeter (3.360 ± 0.1391 mm to 3.249 ± 0.2290 mm). The Feret diameter (1.035 ± 0.0436 mm to 0.996 ± 0.0664 mm) and the minimum Feret diameter (0.944 ± 0.0433 mm to 0.902 ± 0.0564 mm) showed a comparable decreasing tendency over time.

In contrast, soaking in Peridot natural mineral (PNM) water consistently yielded lower values for all measured parameters than in tap water. The projected area decreased from 0.628 ± 0.776 mm^2^ at 5 min to 0.588 ± 0.055 mm^2^ at 30 min. The other values were likewise lower across all time points, with a gradual decline observed during the soaking period. The perimeter values decreased from 3.101 ± 0.2180 mm to 2.962 ± 0.1365 mm. The Feret values range from 0.956 ± 0.0668 mm to 0.916 ± 0.0451 mm, and the minimum Feret values range from 0.862 ± 0.0559 mm to 0.836 ± 0.0437 mm.

Overall, the image analysis demonstrated measurable time-dependent changes in pellet geometry, with more pronounced reductions observed in PNM water than in tap water.

**Figure 5:**
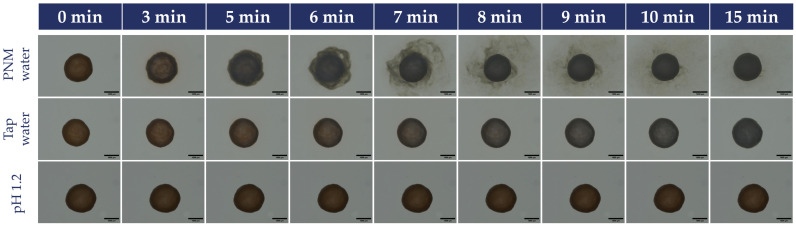
Visual comparison of the individual pellets in PMM water, tap water, and pH 1.2 medium.

Images of the individually inspected pellets are shown in Figure 5. It is visible, that in Peridot (PNM) water, the coating on the pellets breaks down spectacularly just after 5 min. In tap water, the yellow color only fades slightly. In contrast, at pH 1.2, no change occurs within 15 min, indicating that the gastro-resistant coating provides adequate protection.

#### 3.4.4. Dissolution Study Results

**Figure 6:**
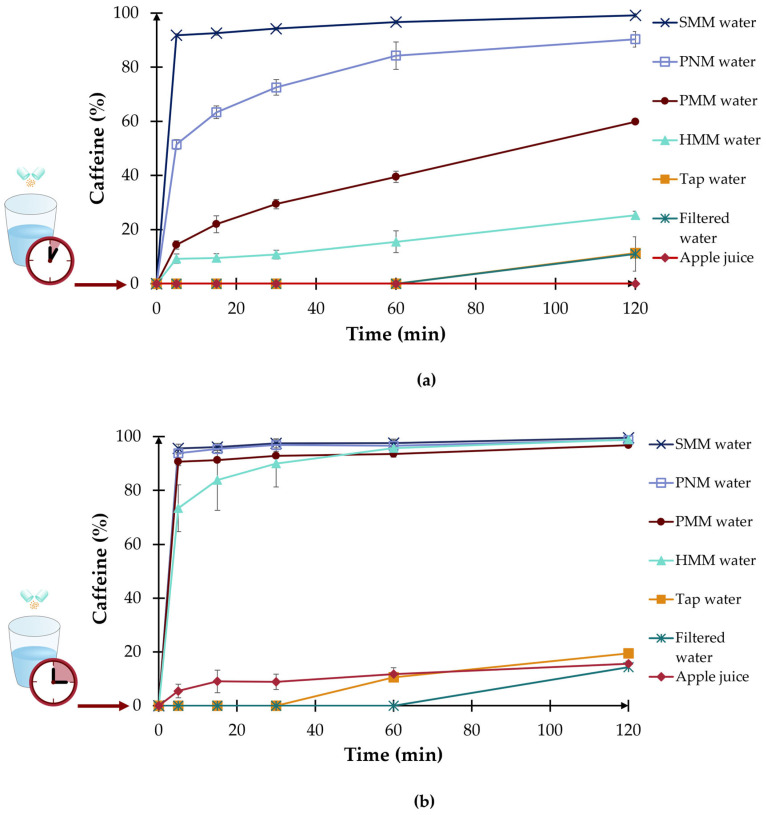

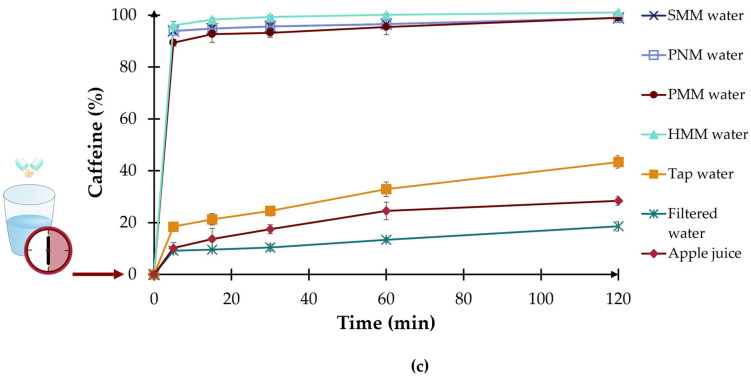
Results of the dissolution test of the API after pre-soaking the pellets in different kinds of media for (**a**) 5 min, (**b**) 15 min, and (**c**) 30 min.

After soaking the pellets in water with different pH values for 5, 15, or 30 min, the dissolution test results varied significantly. The differences in the dissolution behavior of the pellets are shown in Figure 6, and the quantitative percentage data are presented in Appendix A, Table A1.

After 5 min of pre-soaking (Figure 6a), in the case of Salvus medicinal mineral (SMM), more than 90% of the API was already dissolved at the 5-min sampling time. For the other medicinal and natural mineral waters, the effect on the pellets is also visible just after 5 min, but lower. For tap and filtered water, the active ingredient was detectable only after 1 h. In the apple juice, the API concentration remained below the LLOQ level.

After 15 min of pre-soaking (Figure 6b), in the case of the alkalizing waters, remarkable changes are visible: almost complete drug release occurs within a short time, whereas moderate increases are observed in tap water and apple juice. Release in filtered water remained limited throughout the test period, although after 2 h, the cumulative release was nearly identical across apple juice, tap, and filtered water.

After 30 min of pre-soaking (Figure 6c), near-complete drug release was observed with the alkalizing medicinal and mineral water, even after 5 min of the dissolution test. Whereas tap water showed a gradual increase in API content, after 2 h, nearly 50% of the API content was detected. Filtered water consistently resulted in the lowest drug release among all tested media.

Overall, the results demonstrate that both the composition of the administration liquid and the duration of pre-soaking noticeably influence the dissolution behavior of the gastro-resistant pellets.

#### 3.4.5. Statistical Analysis Evaluation

One-way ANOVA indicated a significant effect of the intake medium on API dissolution after 120 min. Dunnett’s post hoc test was applied to compare the dissolution profiles of the pre-soaked samples with those of the samples soaked in tap water, used as the reference. There are significant differences between almost all tested media and tap water, except for filtered water at the 5-min pre-soaking time point (*p* = 0.00193), where the results did not differ significantly, and apple juice at 5 min, where API concentrations remained below the LLOQ and statistical comparison was therefore not performed. The statistical values are shown in Table 9.

## 4. Discussion

The preferred vehicles used for the administration of oral dosage forms can vary widely, especially in children [51]. This also addresses a clinically relevant but underexplored aspect of excipient and vehicle incompatibility, as well as the effect of oral dosage form manipulation on the API.

The preliminary review of 103 SmPCs revealed that in 42 SmPCs, the vehicle was not specified at all, and a further 31 allowed administration with an unspecified liquid. The manipulation of gastro-resistant dosage forms is permitted in 14 capsule and 3 tablet dosage forms. The possibility of this problem is also demonstrated by the fact that it showed considerable variability in the level of guidance provided regarding the appropriate vehicle for administration. Therefore, patients may use medicinal and natural mineral waters, as well as other beverages with different pH and ionic composition. Only 9 SmPCs and PILs provided clearly defined instructions, specifying suitable vehicles such as acidic fruit juice, apple juice, or slightly acidic liquids, and in all 9 cases, the dosage form was manipulable. This widespread lack of specificity in administration guidance is concerning, particularly given that patients with acid reflux or dysphagia—who are the most likely to manipulate their dosage forms—may routinely consume alkalizing mineral waters to relieve gastric symptoms, potentially compromising the enteric coating before ingestion. The vehicles used for ingestion of the medicines are quite varied [52], which can increase the potential for excipient-vehicle incompatibility. In clinical practice, capsule opening and pellet dispersion in liquids are frequently recommended to facilitate administration [53,54]. Moreover, medication-related errors are common in elderly care; most are identified during the preparation and administration phases of the medication process, thereby reducing the effectiveness of the required treatment [55,56].

The appropriate coating level, particularly for a manipulable dosage form of gastro-resistant multiparticulate pellets, is essential. From a practical perspective, our findings emphasize that even small variations in coating level may significantly impact the formulations, especially under non-standard administration conditions. Therefore, our findings, that the 10% weight gained coating was not sufficient, although the 15% *w*/*w* weight gained was satisfactory, was a crucial point in our study.

The in vitro dissolution studies demonstrated a clear time- and medium-dependent effect on gastro-resistant coating integrity. Following only 5 min of pre-soaking in Salvus medicinal mineral water, more than 90% of the API was released already at the 5-min dissolution sampling point, indicating near-complete coating failure. Prolonged pre-soaking (15 and 30 min) further increased this premature API release also in Peridot natural mineral water, Parádi and Hunyadi medicinal mineral water. After 30 min of pre-soaking, near-complete drug release was observed in Salvus, Parádi and Hunyadi medicinal mineral, and in Peridot natural mineral water. In contrast, tap water and filtered water had a markedly lower impact, with filtered water consistently showing the lowest drug release across all pre-soaking durations. From these dissolution studies, it is also evident that not only pH determines coating stability, but also the ion composition of the different liquids [57,58]. It should be acknowledged, that the static pre-soaking model used does not fully replicate the complexity of in vivo gastric conditions, where emptying occurs rapidly and luminal pH changes dynamically. 

These observations were further supported by the testing of marketed gastro-resistant products. It was observed that the gastro-resistant coating resists tap water for 30 min, except for product 3. In contrast, in Salvus medicinal (SMM) and Peridot natural mineral (PNM) water, the coating breaks down within 15 min, but mostly under 5 min, and the waters’ opalescence increases over time, indicating that the coating cannot withstand these conditions [59,60].

The image analysis provided additional quantitative evidence by showing a time-dependent decrease in geometric parameters after soaked in water. Film coating performance is known to depend on environmental exposure and excipients [61], as polymeric films may undergo structural changes in response to the medium composition [62,63]. In tap water, the measured parameters decreased only slightly, consistent with limited surface erosion. In contrast, for PNM, even after 5 min the values were reduced so after a few minutes, only the inert core was measured. Image analysis is a well-established tool in pharmaceutical research and development, providing information on particle morphology, size, shape, and surface characteristics throughout production quality assessment [64,65], offering nondestructive, rapid evaluation [66,67]. 

The clinical relevance of these findings is particularly significant for vulnerable populations, including elderly and dysphagic patients, in whom dosage form manipulation is most commonly practiced [68]. Gastro-resistant coatings are applied to a broad and heterogeneous range of active pharmaceutical ingredients, including proton pump inhibitors, non-steroidal anti-inflammatory drugs and in many of these cases, protection against gastric degradation is essential to preserve drug stability or to minimize gastric irritation. Although dosage form modification can be explicitly permitted in the PILs, the conditions are not specified clearly, or alterations in administration practice may also occur without permission.

## 5. Conclusions

The review of PILs and SmPCs revealed substantial gaps in administration guidance for gastro-resistant oral dosage forms. In 42 cases, the vehicle for administration was entirely unspecified; only 8.7% of the SmPCs examined provided the patient with clear instructions regarding the vehicle for drug administration (amount and type). These findings highlight a regulatory gap that may directly affect therapeutic outcomes, particularly for patients with dysphagia who require dosage form manipulation.

The physicochemical characterization of commonly available beverages demonstrated wide variability in pH and conductivity. Observations from marketed gastro-resistant products confirmed these findings: coating breakdown was visible within 15 min, but mostly within 5 min, upon exposure to Salvus medicinal mineral water and Peridot natural mineral water; tap water preserved coating integrity for the permitted soaking duration, except for product 3. Image analysis further quantified these effects, demonstrating a time-dependent reduction in particle geometric parameters in alkaline medicinal mineral and natural water, indicating progressive coating erosion down to the inert core.

In vitro dissolution studies showed that pre-soaking gastro-resistant Eudragit L 30 D-55-coated pellets in alkalizing medicinal mineral waters and natural medicinal water for just 15 min resulted in more than 90% premature API release upon subsequent exposure to acidic dissolution medium, indicating near-complete coating failure. In contrast, filtered water consistently produced the lowest drug release across all time points. These results confirm that both the pH and the ionic composition of the administration liquid are critical determinants of coating stability.

Collectively, these findings underscore the need for more precise and explicit guidance in SmPCs regarding suitable liquids for the administration of manipulated gastro-resistant products. Recommending slightly acidic or pH-neutral liquids and discouraging the use of alkalizing mineral waters may significantly reduce the risk of unintended premature drug release and improve patient safety, particularly in elderly and dysphagic populations.

## Figures and Tables

**Table 1:** Abbreviations of the liquids used in further experiments.

Liquid	Abbreviation
Salvus medicinal mineral water	SMM
Peridot natural mineral water	PNM
Parádi medicinal mineral water	PMM
Hunyadi János medicinal mineral water	HMM

**Table 2:** The 22 vehicles and the manufacturers.

	Vehicles	Manufacturer/Bottler
Water	Tap water	-
	Filtered water	Culligan Hungary Kft., Budapest, Hungary
	Bottled water—slightly enriched with carbon dioxide	Magyarvíz Kft., Lajosmizse, Hungary
	Salvus medicinal mineral water	Salvus Kft., Pilisszentiván, Hungary
	Peridot natural mineral water	Peridot Aqua Kft., Budapest, Hungary
	Parádi medicinal mineral water	Medaqua Kft., Gyöngyös, Hungary
	Hunyadi János medicinal mineral water	Medaqua Kft., Gyöngyös, Hungary
	Ferenc József medicinal mineral water	Medaqua Kft., Gyöngyös, Hungary
	Mira medicinal mineral water	Medaqua Kft., Gyöngyös, Hungary
	Rajec natural mineral water—sage	B-Beverages Zrt., Székesfehérvár, Budapest
Beverage	Apple juice	Sonny Kft., Budapest, Hungary
	Ice Tea—lemon	Coca-Cola HBC Magyarország Kft, Dunaharszti, Hungary
	Cola Zero	Coca-Cola HBC Magyarország Kft, Dunaharszti, Hungary
	Dry white wine	Matias Borház Kft, Ócsárd, Hungary
Coffee and Tea	Capsuled coffee	Tschibo Budapest Kft., Budapest, Hungary
	Green tea	Orbico Hungary Kft., Bratislava, Slovakia
	Black tea	Orbico Hungary Kft., Bratislava, Slovakia
	Fruit tea	Orbico Hungary Kft., Bratislava, Slovakia
Milk	Alkalizing tea	Oriental Herbs Kft., Budapest, Hungary
	UHT 2.8% milk	Alföldi Tej Kft., Székesfehérvár, Hungary
	Lactose-free milk 1.5%	Alföldi Tej Kft., Székesfehérvár, Hungary
	Almond plant-based drink	Alpro Ltd., Ghent, Belgium

**Table 3:** Fluidized bed bottom-spray parameters.

Manufacturing Parameter	Drug Layering	Film Coating
Fill Weight (g)	200.0	100.0
Nozzle Diameter (mm)	0.8	0.8
Inlet Air Temperature (°C)	45.0	40
Outlet Air Temperature (°C)	37–42	37–40
Spray Air Pressure (bar)	0.8	0.8
Fluid Air Flow Rate (m^3^/h)	80–100	80–100
Feed Rate (g/min)	2–4	2–4
Drying Temperature (°C)	45	40
Drying Time (min)	15	15

**Table 4:** The film-coating dispersion composition.

Component	Function	%; *w*/*w*
Eudragit L 30 D-55	Film-forming polymer	33.30
TEC	Plasticizer	2.00
Talc	Anti-adhesion agent	7.50
Deionized Water	Dispersion medium	57.20

**Table 5:** Meaning of the ATC codes, and the number of preparations belonging to them.

ATC Code	Name	No.
A	Alimentary tract and metabolism	52
*A02*	*Drugs for acid related disorders*	*39*
*A03*	*Drugs for functional gastrointestinal disorders*	*2*
*A06*	*Drugs for constipation*	*2*
*A07*	*Antidiarrheals, intestinal antiinflammatory*	*4*
*A09*	*Digestives, incl. Enzymes*	*4*
*A16*	*Other alimentary tract and metabolism products*	*1*
B	Blood and blood forming organs	9
*B01*	*Antithrombotic agents*	*8*
*B03*	*Antianemic preparations*	*1*
C	Cardiovascular system	1
*C05*	*Vasoprotectives*	
J	Antiinfectives for systemic use	5
*J02*	*Antimycotics for systemic use*	
L	Antineoplastic and immunomodulating agents	22
*L04*	*Immunosuppressants*	
M	Musculo-skeletal system	1
*M01*	*Antiinflammatory and antirheumatic products*	
N	Nervous system	11
*N03*	*Antiepileptics*	*1*
*N06*	*Psychoanaleptics*	*10*
R	Respiratory system	2
*R05*	*Cough and cold preparations*	*1*
*R06*	*Antihistamines for systemic use*	*1*

**Table 6:** Viscosity values of the seven vehicles used for the dissolution study.

Shear Rate [s^−1^]	Viscosity [mPa.s]						
	SMM	PNM	PMM	HMM	Tap Water	Filtered Water	Apple Juice
498–508	1.0	0.9	0.9	1.0	0.9	0.9	1.1
691–713	1.0	0.9	0.9	1.1	0.9	0.9	1.2
990–1001	1.0	0.9	0.9	1.0	0.9	0.9	1.2
1396–1427	1.0	0.9	0.9	1.0	0.9	0.9	1.1
1991–2057	1.0	0.9	0.9	1.0	0.9	0.9	1.1
Average	1.0 ± 0.01	0.9 ± 0.01	0.9 ± 0.01	1.0 ± 0.02	0.9 ± 0.00	0.9 ± 0.01	1.1 ± 0.01

**Table 7:** The validation parameters.

Samples (mg/100 mL)	Accuracy (%)	Precision (RSD%)	Absorbance
QC1 0.075	103.32	3.43	0.0414 ± 0.00134
QC2 0.1	102.52	3.20	0.0541 ± 0.00166
QC3 0.75	100.55	0.18	0.3837 ± 0.00069

**Table 8:** The results of the image analysis (area, perimeter, Feret, Feret min), after leaving the pellets in tap water and Peridot natural mineral (PNM) water for different time periods.

Soaking Time (min)		Area (mm^2^)	Perimeter (mm)	Feret (mm)	Feret Min (mm)
PNM water	5	0.628 ± 0.0777	3.101 ± 0.2180	0.956 ± 0.0668	0.862 ± 0.0559
	10	0.597 ± 0.0654	2.997 ± 0.1637	0.926 ± 0.0506	0.840 ± 0.0499
	15	0.597 ± 0.0594	2.980 ± 0.1519	0.920 ± 0.0472	0.844 ± 0.0443
	20	0.599 ± 0.0785	3.007 ± 0.2106	0.931 ± 0.0553	0.841 ± 0.0797
	25	0.592 ± 0.0597	2.962 ± 0.1483	0.920 ± 0.0464	0.836 ± 0.0468
	30	0.588 ± 0.0548	2.962 ± 0.1365	0.916 ± 0.0451	0.836 ± 0.0437
Tap water	5	0.750 ± 0.0609	3.360 ± 0.1391	1.035 ± 0.0436	0.944 ± 0.0433
	10	0.747 ± 0.0724	3.366 ± 0.1688	1.035 ± 0.0558	0.941 ± 0.0490
	15	0.727 ± 0.0735	3.336 ± 0.1838	1.023 ± 0.0561	0.932 ± 0.0474
	20	0.730 ± 0.0656	3.340 ± 0.1751	1.022 ± 0.0472	0.932 ± 0.0469
	25	0.697 ± 0.0807	3.276 ± 0.2032	1.004 ± 0.0612	0.910 ± 0.0544
	30	0.683 ± 0.0798	3.249 ± 0.2290	0.996 ± 0.0664	0.902 ± 0.0564
Coated pellets		0.711 ± 0.0648	3.289 ± 0.1617	1.008 ± 0.0467	0.922 ± 0.0491

**Table 9:** *p*-values for the dissolution study after 120 min, after 5, 15, and 30 min pre-soak.

Media	5 min	15 min	30 min
Apple juice–Tap water	NA	*p* < 0.001	*p* < 0.001
Filtered water–Tap water	*p* = 0.00193	*p* < 0.001	*p* < 0.001
HMM–Tap water	*p* < 0.001	*p* < 0.001	*p* < 0.001
PMM–Tap water	*p* < 0.001	*p* < 0.001	*p* < 0.001
PNM–Tap water	*p* < 0.001	*p* < 0.001	*p* < 0.001
SMM–Tap water	*p* < 0.001	*p* < 0.001	*p* < 0.001
SNM–Tap water	*p* < 0.001	*p* < 0.001	*p* < 0.001

NA: Value below the LLOQ; statistical comparison with tap water was therefore not performed.

## Data Availability

The data presented in this study are openly available in the article.

## Abbreviations

The following abbreviations are used in this manuscript:

API	Active Ingredient
HMM	Hunyadi János medicinal mineral water
HPMC	Hydroxypropyl Methylcellulose
LLOQ	Lower Limit of Quantification
MUPS	Multiple unit pellet systems
PIL	Patient Information Leaflet
PMM	Parádi medicinal mineral water
PNM	Peridot natural mineral water
RSD	Relative Standard Validation
SMM	Salvus medicinal mineral water
SmPC	Summary of Product Characteristics
TEC	Triethyl Citrate
QC	Quality Control

## Appendix A

Table A1 presents the quantitative dissolution data expressed as the mean ± standard deviation (*n* = 3) for all tested media across the three pre-soaking durations (5, 15, and 30 min) and three dissolution sampling timepoints (5, 60, and 120 min). The data confirm that alkalizing medicinal and natural mineral waters (SMM, PNM, PMM, HMM) induced the highest premature drug release across all pre-soaking durations and sampling timepoints. Tap water and filtered water consistently produced markedly lower drug release, with filtered water showing the lowest values throughout. Apple juice resulted in negligible drug release at the 5-min pre-soaking time point, with moderate release observed after longer pre-soaking durations. These data underlie the statistical comparisons reported in the main text and serve as a comprehensive reference for the full dissolution profiles across all tested conditions.

**Table A1:** The % results of the dissolution study in different media and pre-soaking time.

Pre-Soaking Time (min)	Dissolution Study Sampling Time (min)	Released Drug (%)						
		SMM Water	PNM Water	PMM Water	HMM Water	Tap Water	Filtered Water	Apple Juice
5	5	91.8 ± 0.66	51.5 ± 1.83	14.3 ± 1.56	9.20 ± 1.77	-	-	-
	60	96.7 ± 0.71	84.3 ± 5.03	39.5 ± 2.07	15.5 ± 4.00	-	-	-
	120	99.2 ± 0.70	90.3 ± 2.88	59.8 ± 0.34	25.3 ± 1.42	11.3 ± 1.5	11.0 ± 6.34	-
15	5	95.6 ± 1.61	93.8 ± 0.31	90.7 ± 1.49	73.4 ± 8.74	-	-	5.5 ± 2.49
	60	97.6 ± 1.46	96.6 ± 1.70	93.6 ± 1.18	95.7 ± 3.01	10.5 ± 0.00	-	11.8 ± 2.33
	120	99.6 ± 1.11	98.8 ± 1.01	96.9 ± 0.55	98.9 ± 1.54	19.5 ± 0.73	14.3 ± 0.43	15.6 ± 0.99
30	5	94.0 ± 1.10	94.2 ± 0.71	89.4 ± 1.36	96.1 ± 1.51	18.4 ± 1.69	9.2 ± 0.00	10.2 ± 2.22
	60	96.6 ± 0.66	98.1 ± 0.09	95.5 ± 3.02	100.1 ± 0.49	32.9 ± 2.80	13.3 ± 0.86	24.6 ± 3.39
	120	99.9 ± 0.75	100.5 ± 0.78	99.0 ± 1.90	100.1 ± 0.40	43.4 ± 2.41	18.6 ± 1.80	28.4 ± 1.76
